# Second-generation bi-cruciate stabilized total knee system has a lower reoperation and revision rate than its predecessor

**DOI:** 10.1007/s00402-018-3019-5

**Published:** 2018-08-25

**Authors:** Bernhard Christen, Branko Kopjar

**Affiliations:** 1 CHRISTENORTHO AG, Schaenzlistrasse 39, 3000 Bern 25, Switzerland; 2 Department of Health Services, University of Washington, Seattle, WA USA

**Keywords:** Total knee arthroplasty, Bi-cruciate stabilized, Guided motion, Complications, Reoperations, Revisions

## Abstract

**Introduction:**

Total knee arthroplasty (TKA) can provide pain relief and good long-term results. However, nearly 30% of post-surgical patients are unsatisfied due to persistent pain and functional deficits. A second-generation bi-cruciate stabilized TKA device has a post-cam mechanism with an asymmetric femoral component, a polyethylene insert, and a medially concave and laterally convex shape. The device is designed to provide guided motion, and thus improve knee kinematics by more closely approximating a normal knee. The aim of this study was to evaluate early complication and revision rates of the second-generation device and to compare its clinical performance to the first-generation device.

**Materials and methods:**

In this retrospective, longitudinal, non-concurrent cohort study, 140 TKAs were performed using the second-generation device on 131 patients from 2012 to 2016, and 155 TKAs were performed using the first-generation device on 138 patients from 2009 to 2012. Primary outcomes were occurrence of revisions and reoperations.

**Results:**

There were 31 reoperations [3.21 per 100 observed component years (OCY)] in 22 (2.28 per 100 OCY) TKAs in the first-generation device cohort compared to five reoperations (1.92 per 100 OCY) in four TKAs (1.54 per 100 OCY) in the second-generation device cohort. The adjusted hazard ratio (HR) was 3.50 (*P* = 0.0254). There were 21 revisions (2.17 per 100 OCY) in 16 (1.66 per 100 OCY) TKAs in the first-generation device cohort, compared to only three revisions (1.15 per 100 OCY) in two TKAs (0.77 per 100 OCY) in the second-generation device cohort. The adjusted HR was 4.16 (*P* = 0.0693).

**Conclusion:**

The improved design of the second-generation device appears to be associated with a lower risk of reoperation and revision compared to that of the first-generation device.

**Level of evidence:**

III.

## Introduction

Total knee arthroplasty (TKA) can offer pain relief and good long-term results [1, 2]. However, up to 30% of patients are not satisfied with the outcome [3–5]. A common complaint is persistent pain [6–8], which is mainly anterior and usually depends on activity. Other than pain, reasons for dissatisfaction with the outcome include functional deficits in daily life [6, 9, 10] as well as unfulfilled patient expectations [11–14].

In most patients, the anterior cruciate ligament (ACL) is missing during TKA surgery. Whether the posterior cruciate ligament (PCL) should be retained or sacrificed is debated. The JOURNEY™ Bi-cruciate Stabilized (BCS) Total Knee System (TKS) (Smith & Nephew, Memphis, TN, USA) replaces both cruciate ligaments. The device is designed to more closely approximate those of a normal knee. It has an asymmetric femoral component, a polyethylene insert replicating 3° of the tibial varus, with a medially concave shape and a laterally slightly convex shape [15].

The function of both the ACL and PCL is replicated by a post-cam mechanism that engages not only posteriorly, but also anteriorly. Cam and post are asymmetrical and guide the tibia in flexion to external rotation in relation to the femur. In full extension, the mechanism creates an internal rotation known as the screw-home mechanism. The goal is to provide a “guided motion” which should lead to kinematics similar to that of a normal knee [16–19]. This guided motion has been reported in different studies comparing the JOURNEY™ I BCS prosthesis to other TKA systems and normal knees [20–25]. In vivo fluoroscopic studies demonstrated that nearly normal kinematic motions can be attained with the JOURNEY™ BCS TKS [21, 22, 24, 26–30]. However, the kinematic profile still differs from that of a normal knee [22].

Despite improved kinematics, a single study of JOURNEY™ I failed to demonstrate superior clinical outcomes in comparison to traditional TKA devices [31]. Most importantly, early complication and revision rates of JOURNEY™ are higher compared to those of standard TKA devices [15, 31–34]. Friction of the iliotibial band due to excessive lateral rollback as well as higher risk of knee dislocation were associated with the use of the JOURNEY™ I system [33, 34]. The re-designed JOURNEY™ II was introduced in 2012, featuring a design modification of the femoral component and the polyethylene insert. One study published in 2017 compared the performance of JOURNEY™ I and JOURNEY™ II, but the follow-up period was brief and certain important variables differed between the two treatment groups [35]. Currently, there are no studies with long-term follow-up describing the clinical performance of JOURNEY™ II.

The aim of this study was to evaluate early complication and revision rates of JOURNEY™ II and to compare its performance to JOURNEY™ I. The hypothesis was that the device modification applied in the JOURNEY™ II BCS device would reduce the risks of reoperation and revision.

## Materials and methods

Ethics Committee approval was obtained for this study. All consecutive cases of TKA using JOURNEY™ I or JOURNEY™ II in a single-surgeon (BC) clinical practice were retrospectively reviewed. JOURNEY™ I was used from December 2006 through July 2012; JOURNEY™ II has been used since September 2012. The JOURNEY™ II sample consisted of 140 consecutive TKAs performed in 131 patients from October 2012 through September 2016. The comparison JOURNEY™ I sample consisted of 155 consecutive TKAs performed in 138 patients from January 2009 through July 2012. Data was available for routine clinical follow-ups at 2, 4, and 12 months postoperatively and thereafter as clinically indicated for any complications.

The primary endpoint of this study was surgical revision of the index TKA. Revision was defined as the replacement of any device component (femoral or tibial component, tibia insert and patellar button) or the addition of a patellar button if one was not implanted during the index surgery. The secondary endpoint was any type of reoperation, whether revision surgery or not.

All knees were operated with a tibia-first surgical technique including imageless intraoperative computer navigation for the tibial and distal femoral osteotomies, using a ligament balancer in full extension and 90° flexion cross-checked by standard bony landmarks.

To replicate normal knee kinematics, the JOURNEY™ II BCS provides more mobility in the lateral compartment than other total knee systems provide. For patients who presented with significant varus (bow-legged) or valgus (knock-kneed) deformities (> 15°), morbid obesity, or deficient collateral ligaments, the surgeon decided if additional implant constraint was appropriate. The flexion space was assessed under full ligament tension (e.g., laminar spreaders) with the patella reduced, and a constrained implant option was kept on hand [36].

### Statistical methods

Incidence rates were estimated as events per 100 observed component years (OCY), which is the time from the index surgery to the event, whether revision or reoperation. Cases without reoperation or revision were censored on September 30, 2016. Confidence intervals for the incidence rates were estimated by mid-p approach. The event-free survivorship was estimated for any reoperations and revisions using the Kaplan–Meier estimator. The differences between the cohorts were tested using a log-rank test. Finally, revision-free and reoperation-free survivals were analyzed using the Cox proportional hazards model adjusting for age, sex, and bilateral surgery. Differences in cumulative hazards were evaluated using adjusted hazard ratio (HR).

## Results

From October 2012 through September 2016, 140 JOURNEY™ II TKAs were performed in 131 patients (80 female, 51 male) with a mean age of 65.0 years (40.7–89.6 years) at the time of the index surgery. Nine patients had bilateral surgery and 73 (52.1%) were left knees only. In 119 TKAs (85.0%), patellae were resurfaced with a standard three-pegged button with a flat backside surface; in 21 TKAs, patellae (15.0%) were not resurfaced.

The comparison cohort consisted of 155 JOURNEY™ I TKAs performed in 138 patients from January 2009 through July 2012 using the same surgical technique. Eighty patients (58%) were females; mean age at the time of the index surgery was 66.7 years (41.3 to 83.1 years). Seventeen patients were operated bilaterally; 74 (49%) were left knees only. In 60 (39.7%) TKAs, patellae were not resurfaced. Of 95 resurfaced patellae, a bi-convex patellar button was used in 27 knees, and the standard design of three pegs and a flat backside surface was used in 68 knees. There were no differences between the cohorts in age, gender, proportion of TKAs that were bilateral, side (left or right), and proportion of TKAs involving patellar resurfacing.

The average length of follow-up in the JOURNEY™ II cohort was 1.86 years (0.05–3.92 years), compared to 6.23 years (4.40–7.70 years) in the JOURNEY™ I cohort. Altogether, there were 260 OCY in the JOURNEY™ II cohort and 966 OCY in the JOURNEY™ I cohort.

Considering multiple reoperations in the same TKAs, there were 31 (3.21 per 100 OCY; 95% CI 2.18–4.55) reoperations in the JOURNEY™ I cohort and five (1.92 per 100 OCY; 95% CI 0.69–4.48) in the JOURNEY™ II cohort (*P* = 0.2894).

Considering TKAs with any (one or more) reoperations, there were 22 cases (2.28 per 100 OCY; 95% CI 1.43–3.45) among 155 TKAs in the JOURNEY™ I cohort, and four cases (1.54 per 100 OCY; 95% CI 0.41–3.94) among 140 TKAs in the JOURNEY™ II cohort (*P* = 0.4945).

**Fig. 1:**
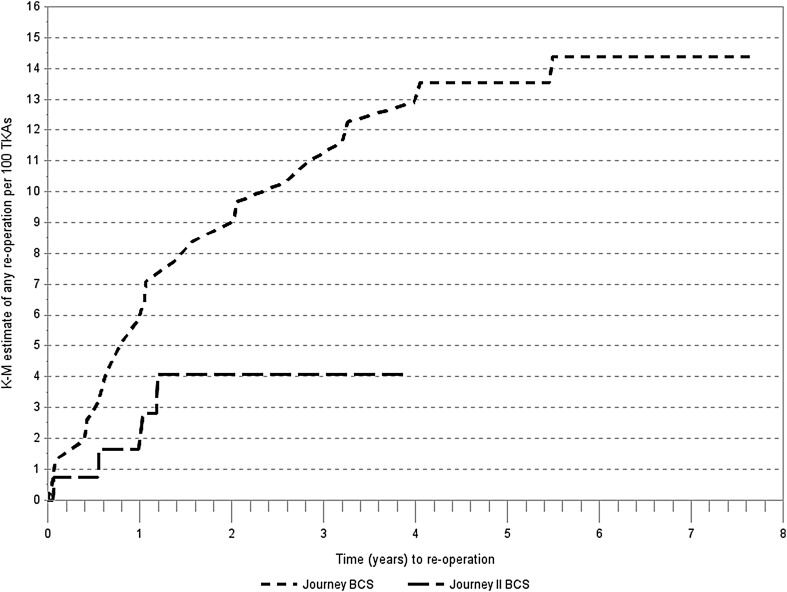
K–M estimate of any reoperation for JOURNEY™ BCS and JOURNEY™ II BCS total knee arthroplasty

Figure 1 shows K–M failure rate estimate of time to first reoperation. The cumulative incidence of reoperations in the JOURNEY™ I cohort with a maximum follow-up of 7.7 years was 14.38 per 100 TKAs, and the incidence in the JOURNEY™ II cohort was 4.06 per 100 TKAs with a maximum follow-up of 3.92 years. Of note, the cumulative incidence in the JOURNEY™ I cohort at 4-year follow-up was about 13 per 100 TKAs. The log-rank test P value for comparison of survivorship between JOURNEY™ I and JOURNEY™ II was 0.0612.

**Fig. 2:**
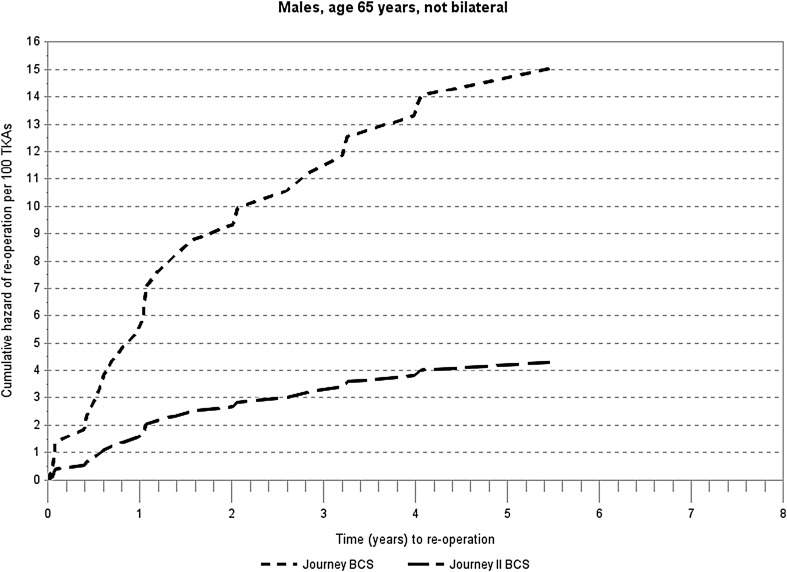
Cox regression cumulative hazard of any reoperation for JOURNEY™ BCS and JOURNEY™ II BCS total knee arthroplasty at covariates males, 65 years of age, not bilateral surgery patient

Table 1 and Fig. 2 show results of the Cox regression modeling of hazard for any reoperation. Relative hazard was 3.50 times higher for JOURNEY™ I TKAs than JOURNEY™ II TKAs (*P* = 0.0258). Age was a significant predictor for reoperation; older patients were less likely to require reoperation compared to younger patients. Gender and bilateral surgery were not associated with reoperation risk.

**Table 1:** Hazard ratio estimates for any reoperation in JOURNEY™ I BCS and JOURNEY™ II BCS total knee arthroplasty

Parameter	Hazard ratio	95% hazard ratio confidence limits		*P*
Age	0.94	0.90	0.98	0.0077
Cohort (JOURNEY I vs. JOURNEY II)	3.50	1.16	10.52	0.0258
Gender (males vs. females)	0.75	0.33	1.66	0.4742
Bilateral (yes vs. no)	0.41	0.12	1.39	0.1533

Considering multiple revisions in the same TKAs, there were 21 (2.17 per 100 OCY; 95% CI 1.35–3.32) revisions in the JOURNEY™ I cohort and three revisions (1.15 per 100 OCY; 95% CI 0.23–3.37) in the JOURNEY™ II cohort (*P* = 0.3094). Considering TKAs with any (one or more) revisions, there were 16 cases (1.66 per 100 OCY; 95% CI 0.94–2.69) among 155 TKAs in the JOURNEY™ I cohort and two cases (0.77 per 100 OCY; 95% CI 0.09–2.78) among 140 TKAs in the JOURNEY™ II cohort (*P* = 0.3120).

**Fig. 3:**
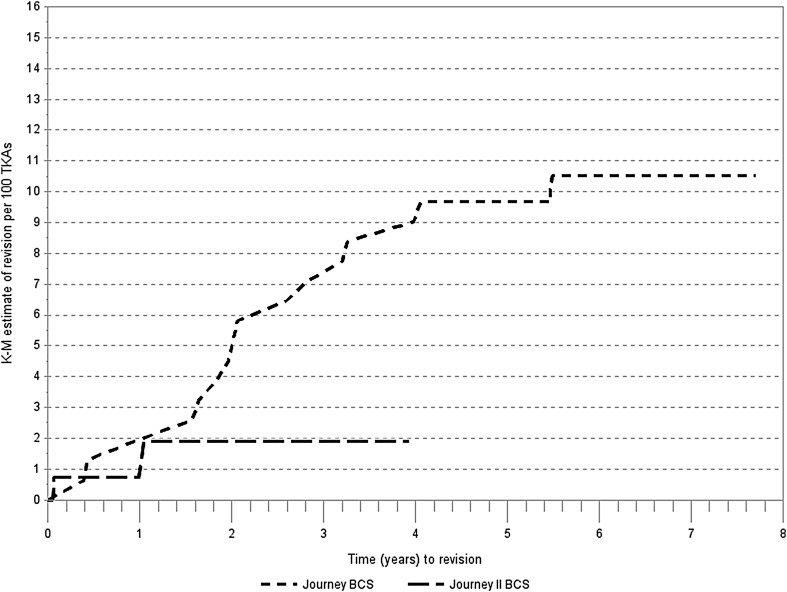
K–M estimate of any revision for JOURNEY™ BCS and JOURNEY™ II BCS total knee arthroplasty

Figure 3 shows K–M failure rate estimate of time to first revision. The cumulative incidence of revisions in the JOURNEY™ I cohort with a maximum follow-up of 7.7 years was 10.51 per 100 TKA, and the incidence in the JOURNEY™ II cohort with a maximum follow-up of 3.92 years was 1.89 per 100 TKAs. Of note, the cumulative incidence in the JOURNEY™ I cohort at 4 years was 9.68 per 100 TKAs. The log-rank test *P* value for comparison of survivorship between JOURNEY™ I and JOURNEY™ II was 0.1156.

**Fig. 4:**
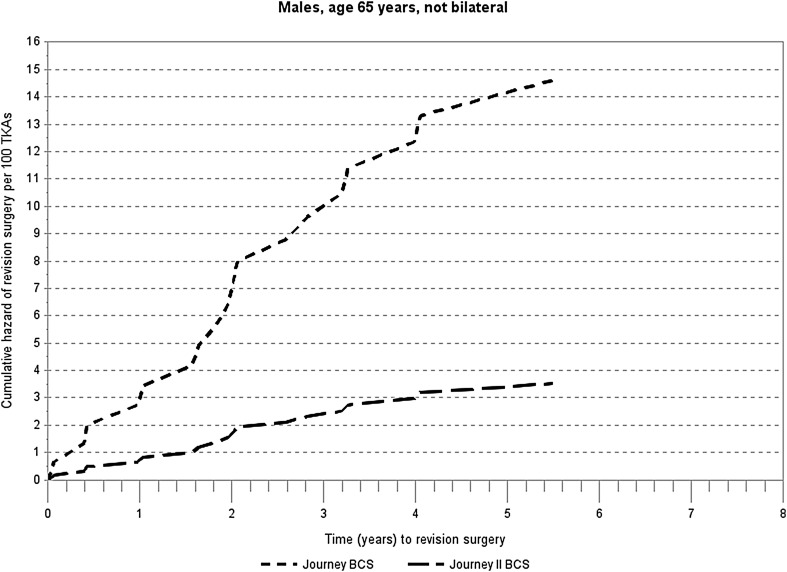
Cox regression cumulative hazard of any revision for JOURNEY™ BCS and JOURNEY™ II BCS total knee arthroplasty

Table 2 and Fig. 4 show results of the Cox regression relative hazard modeling for the first revision. The relative hazard was 4.16 times higher for JOURNEY™ I than JOURNEY™ II TKAs (*P* = 0.0693). Age was a significant predictor of revision; older patients were less likely to require revision compared to younger patients. Gender, bilateral surgery, and patellar resurfacing were not associated with revision risk.

**Table 2:** Hazard ratio estimates for revision in JOURNEY™ I BCS and JOURNEY™ II BCS total knee arthroplasty

Parameter	Hazard ratio	95% hazard ratio confidence limits		*P*
Age	0.94	0.89	0.99	0.0254
Cohort (JOURNEY I vs. JOURNEY II)	4.16	0.89	19.34	0.0693
Gender (males vs. females)	1.34	0.52	3.43	0.5443
Bilateral (yes vs. no)	0.72	0.20	2.52	0.6134
Patella resurfacing (yes vs. no)	1.27	0.44	3.64	0.6620

Of 16 revised TKAs in the JOURNEY™ I cohort, 11 (68.75%) were total revisions and five (31.25%) were partial revisions (three patellar button replacements and one tibia insert replacement). Of two revised TKAs in the JOURNEY™ II cohort, there was one case of total revision that occurred at 3 weeks following the index surgery and one case of patella replacement at 12 months after the primary implantation. The reasons for revision in the JOURNEY™ I cohort were infection (four cases), instability (four cases), peri-prosthetic fracture (three cases), aseptic loosening (one case), iliotibial band (ITB) friction syndrome (one case), dislocation (two cases), and pain (one case). One revision in the JOURNEY™ II cohort was due to the trauma-related peri-prosthetic femoral fracture and the second revision was due to patellar fracture and necrosis in the resurfaced patella.

In the JOURNEY™ I cohort, there were 5/56 (8.93%) revisions in non-resurfaced TKAs and 11/99 (11.11%) revisions in resurfaced TKAs. In the JOURNEY™ II cohort, there were no revisions among 21 non-resurfaced TKAs and 2/119 (1.68%) revisions in resurfaced TKAs.

## Discussion

The study hypothesis was confirmed. JOURNEY™ II has a lower risk of reoperation and a lower risk of revision compared to JOURNEY™ (I). The risk of revision was found to be 4.2 times higher and the risk of reoperation was 3.5 times higher for JOURNEY™ I than JOURNEY™ (II). Overall, the cumulative revision risk in JOURNEY™ II was 1.89 per 100 TKAs at 4 years, which compares favorably with standard total knee systems.

This is the first study with long-term follow-up that compares the clinical performance of both the older and newer versions of the JOURNEY™ BCS TKS, taking into consideration the risks of reoperation and revision. While the high revision rate of JOURNEY™ I raised concerns, it appears that the device modification applied in JOURNEY™ II was successful in reducing excessive revision risk.

The goal of TKA has historically been restoration of neutral mechanical alignment. Complicating this goal is that patients presenting with osteoarthritis often have either varus or valgus deformity. Significant deformities are more difficult to correct to neutral mechanical alignment if conventional instrumentation is used [37, 38]. Depending on the degree of deformity, the surgical technique, and the choice of implant, the amount of necessary constraint might differ [38, 39]. Thienpont et al. conducted bone morphotype analysis on 96 patients with varus or valgus alignment who underwent TKA. Bone morphotype analysis provides better understanding of the deformity level, allows the surgeon to estimate how much deformity correction can be obtained, helps the surgeon decide if intra-articular correction is possible, and helps with planning of ligamentous release and the amount of constraint to choose. Fifty patients had one osteoarthritic and one non-arthritic knee, and both knees had the same type of varus or valgus alignment. The controls were 46 patients with only ligamentous problems, and both knees had neutral alignment. The authors found that bone morphology in varus and valgus deformity is different before and after OA, and that perpendicular cuts to mechanical axes do not necessarily lead to a neutral mechanical axis. Deformities over 10° usually had an extra-articular component, thus correction to neutral alignment with conventional instruments might not be sufficient to compensate for femoral bowing or extra-articular deformity [38]. The JOURNEY™ II BCS provides more mobility in the lateral compartment than other total knee systems provide, to replicate normal knee kinematics. For patients who presented with significant varus or valgus deformities (> 15°), morbid obesity, or deficient collateral ligaments, the surgeon decided if additional implant constraint was appropriate. The flexion space was assessed under full ligament tension (e.g., laminar spreaders) with the patella reduced, and a constrained implant option was kept on hand [36].

Minoda et al. investigated how the design of the post-cam mechanism affects the risk of impingement of the patellar component on the tibial post (patella post impingement, PPI) during deep flexion after TKA. PPI can cause anterior knee pain, increased patellofemoral pressure, wear of the polyethylene patellar component and tibial post, and reduced range of motion [40]. Using navigation, five posterior-stabilized total knee prostheses were implanted. The tibial component was 10 mm thick in all prostheses; patellar tendon length was represented by the tibial-patellar clearance (TPC), which varied from 18 to 40 mm. The authors found that the tibial-patellar clearance (TPC) and the design of the tibial post both affected the PPI after posterior-stabilized TKA. A longer tibial-patellar clearance (TPC), which is affected by the amount of bone resected from the proximal tibia and distal femur, resulted in a larger PPI angle. Furthermore, a larger tibial post (as in constrained prostheses) resulted in a smaller PPI angle, meaning that the anterosuperior part of the tibial post impinged on the patellar component during deep flexion. To maintain an appropriate TPC and avoid PPI, the authors recommended that surgeons adjust the amount of bone resected from the proximal tibia and distal femur during TKA [41]. The second-generation iteration of the JOURNEY™ BCS addresses this risk of impingement. The cam in the JOURNEY™ II BCS has been re-positioned superior to the original location and thus decreases femoral rollback in the targeted ranges of motion, increases femoral external rotation, and lowers the point of tibial post contact in deep flexion. The tibial post is now anterior to the original location and the height increased by taller PS femoral box walls. The new post position further maintains more anatomically correct femoral rollback, thus reducing iliotibial band (ITB) and iliotibial-patellar band (ITPB) tension. In addition, the tibial post height balances patellar impingement in flexion, while maximizing the dislocation safety factor [42].

Hommel and Wilke assessed differences between JOURNEY™ I and JOURNEY™ II in 257 patients with osteoarthritis of the knee [35]. The first 153 patients received JOURNEY™ I and the remaining 104 patients received JOURNEY™ II. All knees were operated with an extension-first surgical technique. The authors found good early functional results and an acceptable rate of complications with both devices. However, the mean follow-up time for JOURNEY™ II patients was brief at 15 months (range 12–18 months), and follow-up times and other variables differed between the groups.

JOURNEY™ II offers an innovative design that better resembles normal knee kinematics compared to traditional knee replacement devices. One study investigated the JOURNEY™ I device; however, the authors were not able to demonstrate that the improved dynamics could translate into improved functional and quality of life outcomes in patients [31].

Murakami et al. compared clinical outcomes and in vivo knee joint kinematics during stair climbing in rotating platform cruciate-retaining (CR) and posterior-stabilized (PS) mobile-bearing TKAs on clinical outcomes and in vivo knee joint kinematics during stair climbing [43]. Outcomes were evaluated in 20 successful TKAs, including ten CR knees and ten PS knees. The parameters investigated included isometric extensor torque, anterior translation, posterior translation, and total external rotation. The authors found that both CR and PS types of rotating platform mobile-bearing TKAs provided reproducible knee joint kinematics during stair climbing and equivalent clinical patient-reported outcomes. In fact, the patient-reported outcomes using the Knee Society Score 2011 (KSS 2011) were generally comparable to those found by Matsuda et al. [2] and Kawahara et al. [44] in their evaluations of fixed-bearing TKAs.

This is the first study with longer-term follow-up that describes patient outcomes of the current JOURNEY™ II BCS device.

This analysis has some limitations. This study was a non-randomized comparison of the reoperation and revision risks of two devices, and the compared cohorts did not begin concurrently. Non-randomized comparisons are subject to confounding that arise from comparing cohorts that possibly differ in characteristics of importance for the measured outcomes. Further, the risk of reoperation and revision increase over time. The JOURNEY™ I cohort had a longer follow-up time than the JOURNEY™ II cohort.

To minimize these limitations, three different statistical approaches were applied to describe and compare the reoperation and revision risks: the person-time incidence rate, Kaplan–Meier non-parametric estimates, and Cox regression analysis. Each of these approaches has its strengths and weaknesses. The strength of the person-time incidence rate is that it provides a simple way to compare two devices using the same, easily understandable statistic. The major limitation is that the approach assumes that the risk is constant over time. Such an assumption is not accurate for TKA as the risk is higher in the first year than in the later years. Since the JOURNEY™ II cohort had a shorter follow-up than the JOURNEY™ I cohort, the incidence rate estimates were biased in favor of the JOURNEY™ I. Despite this bias, the comparable person-time statistics favored JOURNEY™ II, although statistical significance was not reached for any of the person-time comparisons. Kaplan–Meier non-parametric estimates overcome this limitation and provide a less-biased comparison. Both K–M estimates favored JOURNEY™ II; the estimate for risk of reoperation reached statistical significance. Due to a larger number, the analysis of reoperations had more statistical power than the analysis of revisions and it is reasonable to assume that the lack of statistical significance for the risk of revision is due to type II error. The limitation of K–M estimates is that they are insensitive to effects of confounders. Cox regression analysis allows for the adjustment for confounders. After the adjustment for confounders, the risk estimates remained substantially lower in the JOURNEY™ II cohort. None of these statistical approaches address possible historical confounding effects due to a non-concurrent study design. Theoretically, it is possible that significant time trends or events have occurred that would bias these results. No major health care advances or changes have occurred that would account for the observed difference. Furthermore, all surgeries were performed by the same surgeon using the same surgical technique. A possible learning curve effect was addressed by excluding the first 108 cases in the JOURNEY™ I cohort. From a surgical perspective, there was no difference between the JOURNEY™ I and JOURNEY™ II surgeries. All available consecutive cases were included in the analysis. Revisions that occurred elsewhere were captured and reported. The compared cohorts were similar in age, gender, and the need for bilateral surgery.

These results have important clinical implications. In short-term follow-up, JOURNEY™ II has a low and acceptable risk of revision. Studies with longer follow-ups and those investigating functional and quality of life outcomes are warranted to fully qualify the safety and effectiveness of JOURNEY™ II BCS total knee system.
